# Catastrophic health expenditure associated with non-inpatient costs among middle-aged and older individuals in China

**DOI:** 10.3389/fpubh.2024.1454531

**Published:** 2025-01-17

**Authors:** Xiaojuan Zhang, Kun Zhu

**Affiliations:** ^1^The Institute of Medical Information, Chinese Academy of Medical Sciences and Peking Union Medical College, Beijing, China; ^2^Chinese Academy of Fiscal Science, Beijing, China

**Keywords:** out-of-pocket healthcare expenditure, catastrophic health expenditure, China, non-inpatient, outpatient

## Abstract

**Background:**

Since their establishment, the two predominant social health insurance schemes in China, Urban Employee Medical Insurance (UEMIS) and Urban and Rural Residents’ Medical Insurance (URRMS), have primarily focused on covering non-inpatient expenditure, while costs associated with outpatient care and pharmaceutical purchases have been largely excluded from the insurance benefit package. This study intends to analyze the distribution of non-hospitalization expenditure and assess resulting financial risks, with an objective to reform the health insurance benefit package by including coverage for non-hospitalization costs.

**Methods:**

The primary data were obtained from the 2018 wave of CHARLS, encompassing a total of 12942 individuals for analysis. Assess the financial risk associated with non-hospitalization expenses through catastrophic health expenditures (CHE) and examine the determinants of CHE using logistic regression analysis.

**Results:**

Over 60% of the participants availed non-inpatient services in the month preceding the investigation. A smaller proportion (14.26 and 14.28% for UEMIS and URRMS enrollee, respectively) utilized outpatient services provided by medical institutions, while a larger proportion (54.20 and 56.91% for UEMIS and URRMS enrollee, respectively) purchased medication from pharmacies. The study reveals a distinct subgroup of participants (8.91 and 6.82% for UEMIS and URRMS enrollee, respectively) who incurs substantial out-of-pocket non-inpatient expenditure, surpassing 1,000 RMB per month. However, reimbursement for non-inpatient expenditures is significantly limited under the two predominant health insurance schemes, and there is minimal disparity in the distribution of non-inpatient expenses before and after insurance reimbursement. The prevalence of CHE resulting from non-inpatient costs was substantial, particularly among participants enrolled in URRMS (25.06%) compared to those enrolled in UEMIS (14.26%). The presence of chronic diseases, advanced age, and limited financial resources are all determinants contributing to the occurrence of CHE.

**Conclusion:**

The incorporation of non-inpatient expenses into China’s fundamental health insurance plan remains a contentious issue, given the limited available evidence. This study presents empirical evidence underscoring the significance of non-inpatient expenditures as a determinant of financial risk, thereby emphasizing the imperative to adjust China’s fundamental health insurance benefit package in order to address risks associated with non-inpatient costs, particularly among individuals with chronic illnesses and limited income.

## Introduction

The attainment of universal health coverage is a key objective within the 2030 Sustainable Development Goals. Mitigating health financial risk constitutes a pivotal facet of achieving this goal (1). In pursuit of this aim, the Chinese government has consistently reform health insurance systems.

In the 1990s, China introduced the Urban Employee Medical Insurance system (UEMIS) specifically targeting employed individuals. Initially influenced by the Urban Employee Pension Insurance, UEMIS adopted a combined account model in which individual account funds were allocated for non-inpatient expenses while pooled funds were designated for inpatient expenses, a practice unprecedented except in Singapore. Subsequently, distinct reimbursement policy was established for outpatient and inpatient medical expenses and continue to be utilized up to the present day. The urban and rural residential medical insurance systems (URRMI), derived from the New Rural Agricultural Cooperative Medical for rural residents and Urban Residential Medical Insurance for unemployed urban residents, also prioritize the reimbursement of inpatient expenditures. In 2018, the reimbursement limit for outpatient fees in Chengdu, a city located in western China, was established at 100 RMB for URRMI and zero RMB for UEMIS. Compared to individuals covered by URRMI, those under UEMIS have the option to utilize their individual account funds for outpatient expenses and purchases at pharmacies, thereby obtaining enhanced coverage for non-inpatient medical costs. In the early 21st century, outpatient expenses did not pose a significant burden, while hospitalization costs emerged as the primary driver of health financial risk. However with the advancement of medical technology and changes in disease patterns, outpatient expenses have been rapidly increasing. The mean expenditure per outpatient visit in hospital has risen from 68.8 RMB in 1998 to 342.7 RMB in 2022 in China, thereby exacerbating the issue of financial risk associated with outpatient costs. The Chinese government has implemented a health insurance reimbursement policy aimed at providing specific coverage for selected chronic diseases; however, the extent of this coverage remains limited. Consequently, non-inpatient expenses persistently remain inadequately insured or uninsured, potentially giving rise to financial vulnerability. Therefore, there is an urgent imperative for additional empirical investigations to fortify support for policy enhancements.

Numerous studies have investigated the health financial risk associated with household total medical expenses, utilizing data from social investigations, without distinguishing between outpatient and inpatient expenditures (2–6). Among these studies, some scholars have conducted additional analyses on the correlation between outpatient service utilization and the occurrence of catastrophic health expenditures (7–9). A limited number of studies have also examined the risks associated with outpatient expenses, particularly in developing countries with inadequate healthcare financing systems. For instance, a study analyzed the catastrophic health expenditure resulting from outpatient expenses for non-communicable disease patients in Indonesia. The outcomes of these studies revealed that the economic burden of out-of-pocket health expenditure was significant for outpatient care, and in some cases, even exceeded that of hospitalization (10–13). Unfortunately, there is a lack of similar studies in China specifically addressing the analysis of CHE resulting from non-inpatient services^.^

As previously delineated, the coverage for outpatient expenses is significantly less comprehensive than that for inpatient costs within China’s health insurance systems. Despite this, there seems to be an inadequate emphasis on the potential risks associated with outpatient care expenditures in China. Similar to research conducted in other nations, studies previously conducted in China have primarily focused on analyzing the CHE associated with inpatient cost or the total household health expenses (14–16). However, within the framework of China’s health insurance system that primarily focuses on inpatient expenses, it is imperative to initially analyze the distribution of outpatient expenditures and potential financial risk as a starting point for further discussion. There is a relative scarcity of articles in China that specifically analyze the financial risk associated with outpatient expenses and the coverage of health insurance for such costs (17). This study tries to investigate the financial burden of non-hospitalized patients who have not utilized inpatient services within 1 year prior to the investigation, but have accessed outpatient care or purchased medications from pharmacies, thereby directly reflecting the financial risks associated with non-hospitalized healthcare expenses. This study can address the insufficiency in catastrophic health expenditure resulting from non-inpatient expenses in China, thereby drawing government attention to the risks associated with non-inpatient expenses and enhancing outpatient security measures.

## Methods

### Data source

China Health and Retirement Longitudinal Study (CHARLS) is a comprehensive interdisciplinary survey project conducted by the National School of Development and institute of Social Science Survey of Peking University. In 2011, 2013, 2015, 2018, and most recently in 2020, CHARLS conducted surveys encompassing a total of 150 counties and 450 communities (villages) across China’s 28 provinces, autonomous regions, and municipalities. The samples were selected using a multistage probability sampling method. In the initial stage, 150 county-level units were randomly chosen with a probability-proportional-to-size (PPS) sampling technique from a sampling frame that included all county-level units except Tibet. The sample was stratified by region and within each region by urban districts or rural counties, taking into account per capita statistics on gross domestic product (GDP). The final sample of 150 counties represented 28 provinces. Our sample focused on the lowest level of government organization, consisting of administrative villages (cun) in rural areas and neighborhoods (shequ or juweihui) in urban areas, which served as primary sampling units (PSUs). Within each county-level unit, we selected three PSUs using PPS sampling and we got 450 communities (18). The five surveys conducted between 2011 and 2020 encompassed a total sample size of 17,697, 18,254, 20,273, 19,816, and 19,395 individuals, respectively. However, due to the impact of the COVID-19 pandemic, it was not feasible to collect data on outpatient service utilization and cost for the latest survey; therefore, this article analyzes data from a previous survey conducted in 2018.

The analysis in this paper exclusively focuses on participants who did not utilize inpatient services within 1 year prior to the investigation. Moreover, given the relatively limited proportion of individuals lacking social health insurance or enjoying government-funded medical care, this study solely encompasses participants covered by URRMI and UEMIS. Key indicators such as outpatient expenses and household consumption expenditures that were missing in some data were excluded from the analysis, resulting in a final sample size of 12,942 participants.

### Catastrophic health expenditure (CHE)

Catastrophic health expenditure is a key indicator for measuring financial risk protection. The measurement of CHE involves two aspects: defining the household resources that can be used to pay for health expenditure, and defining the standard of CHE. The measurement of CHE can vary depending on the definition of households’ ability to pay for healthcare. One is budget share approach which defines healthcare affordability as a certain percentage of a household’s total income or total consumption. As is common in most studies, we employ household consumption as a measure of affordability due to its greater reliability in reflecting welfare compared to income (19). This approach does not consider the family’s allocation decisions between essential and non-essential expenditures, thereby lacking the ability to differentiate between populations solely capable of affording necessary expenses and those who possess greater wealth (20). The alternative approach defines healthcare affordability as the residual financial resources available after deducting necessary household expenses, thereby ensuring that household resources allocated for essential expenditures are not diverted towards healthcare services. This approach assumes that low-income households allocate a higher proportion of their earnings towards essential expenses compared to those who are more affluent, thereby addressing the limitation of the budget share approach. However, defining non-essential expenditure poses a challenge and food expenses are generally considered indispensable for families (21). The criteria for CHE are undoubtedly subjective. The budget share approach typically employs a standard of 10 and 25% (22–24), whereas the alternative approach commonly utilizes a standard of 25 and 40% (25–27). In our study, we employ the second approach by utilizing household expenditure and food expenses as primary indicators of household financial resources and essential expenditure, while considering 40% as the threshold criterion.

CHARLS collected household expense data on a weekly, monthly, and annual basis. To calculate the total household expenses, the weekly and monthly expenses were multiplied by 52 and 12 respectively, followed by summing up the weekly, monthly, and annual expenditures. CHARLS investigated the cost in outpatient and pharmacies a month before the survey, which were multiplied by 12 to estimate the annual expenditure on outpatient visits and medication purchases.

In general, CHE is analyzed at the household level. However, due to fragmented outpatient and medication purchasing expenses, it is usually not possible to collect a family’s annual expenses at outpatient and pharmacies. Therefore, only individual’s outpatient expenses in the month before the survey were collected. This study adopts the approach from other studies and analyzes CHE at the individual level by dividing the household non-essential expenditure by the number of family members (22).

Let T represents the health expenditures of a patient, X represent the per capita non-essential household expenses, and Z represent the standard for CHE. If T/X > Z, it is considered that CHE has occurred. The incidence of CHE (H) is denoted as the ratio of individuals who have encountered CHE to the total sample size (Equation 1).


(1) $$H=\frac{1}{N}\overset{N}{\underset{i=1}{\sum }}E_{i}$$

*N*: total sample size; *E_i_*: if Ti/Xi>Z, *E* = 1, else *E* = 0.

### Statistical analysis

Proportions were utilized to describe categorical variables, followed by a chi-square test. Binary logistic multivariate regression analysis was employed to examine the association between individuals’ demographic characteristics and the likelihood of experiencing CHE due to non-inpatient expenditure. The results are presented as odds ratios (OR) with their corresponding 95% confidence intervals and *p*-values.

## Results

### Analysis of demographic characteristics

The demographic characteristics of the study participants are presented in Table 1. A majority of the participants were female (52.53%), married (87.74%), aged between 45 and 60 years old (51.17%), had no formal education (41.49%), resided in rural (72.17%) and eastern area (34.01%) and were covered by URRMI (85.91%).

**Table 1:** Social demographic characteristics of the study population.

Variables	*n*	%
Total	12,942	100
Gender		
Male	6,144	47.47
Female	6,798	52.53
Age		
45–60	6,623	51.17
60–69	4,096	31.65
> = 70	2,223	17.18
Marriage		
With spouse	11,355	87.74
Without spouse	1,587	12.26
Education		
No formal education	5,369	41.49
Primary school	2,868	22.16
Middle school	4,436	34.28
College and above	269	2.08
Type of place of residency		
Urban	3,602	27.83
Rural	9,340	72.17
Area		
Eastern	4,402	34.01
Central	4,307	33.28
Western	4,233	32.71
Health insurance		
UEMIS	1,823	14.09
URRMI	11,119	85.91

### Service utilization

The utilization of outpatient services (14.26 and 14.28% for individuals covered by UEMIS and URRMI respectively) was significantly lower compared to the procurement of medications from drug stores (54.20 and 56.91% for individuals covered by UEMIS and URRMI respectively), as presented in Table 2. As previously mentioned, within the policy context of China, a substantial number of patients choose to purchase medications from pharmacies instead of seeking outpatient services provided by medical institutions to save money.

**Table 2:** Utilization of outpatient and pharmacy services among individuals covered by different health insurance schemes (n/%).

Service utilization	Health insurance schemes	
	UEMIS	URRMI
Total	1823 (100)	11,119 (100)
Monthly outpatient visits		
0	1,563 (85.74)	9,531 (85.72)
1	162 (8.89)	805 (7.47)
2	49 (2.69)	403 (3.49)
Three times and more	49 (2.69)	380 (3.31)
Procurement of medications from drug stores		
Yes	988 (54.20)	6,328 (56.91)
No	835 (45.80)	4,791 (43.09)
Utilization of outpatient services or procurement of medications		
Yes	1,089 (59.74)	6,828 (61.41)
No	734 (40.26)	4,291 (38.59)

### Non-inpatient care expenditure

The percentage of monthly total non-inpatient expenses below 100RMB is 42.22% for UEMIS and 49.88% for URRMI, representing the highest proportions within their respective groups. In terms of outpatient fees, UEMIS demonstrates the highest proportion in the range of 100-300RMB at 22.57%, while for URRMI it is slightly higher at 26.91%. Notably, expenses below 100RMB dominate in pharmacy expenditures.

The proportion of low-cost group of out-of-pocket non-inpatient expenditure (e.g., below 100RMB) is relatively lower for UEMIS (43.59%) compared to URRMI (52.41%). Conversely, the proportion of high-cost groups (e.g., exceeding 1,000 yuan) is higher among UEMIS at a rate of 8.91% compared to URRMI (6.82%) (Table 3).

**Table 3:** Expenditure of non-inpatient service before and after health insurance reimbursement.

Cost categorization (RMB)	Total health expenditure (*n*/%)						Out of pocket health expenditure (*n*/%)					
	Outpatient and pharmacies		Outpatient		Pharmacies		Outpatient and pharmacies		Outpatient		Pharmacies	
	UEMIS	URRMI	UEMIS	URRMI	UEMIS	URRMI	UEMIS	URRMI	UEMIS	URRMI	UEMIS	URRMI
0–100	453 (42.22)	3,353 (49.88)	48 (18.68)	447 (27.97)	483 (49.74)	3,662 (58.79)	367 (43.59)	3,441 (52.41)	69 (30.8)	528 (34.49)	594 (61.17)	3,821 (61.25)
100-	270 (25.16)	1712 (25.47)	58 (22.57)	430 (26.91)	264 (27.19)	1,584 (25.43)	226 (26.84)	1,654 (25.19)	53 (23.66)	398 (26)	208 (21.42)	1,514 (24.27)
300-	107 (9.97)	577 (8.58)	38 (14.79)	220 (13.77)	102 (10.5)	456 (7.32)	78 (9.26)	523 (7.97)	28 (12.5)	179 (11.69)	84 (8.65)	428 (6.86)
500-	131 (12.21)	547 (8.14)	50 (19.46)	242 (15.14)	87 (8.96)	354 (5.68)	96 (11.4)	500 (7.61)	32 (14.29)	221 (14.44)	61 (6.28)	322 (5.16)
1,000-	112 (10.44)	533 (7.93)	63 (24.51)	259 (16.21)	35 (3.6)	173 (2.78)	75 (8.91)	448 (6.82)	42 (18.75)	205 (13.39)	24 (2.47)	153 (2.45)
Total	1,073	6,722	257	1,598	971	6,229	842	6,566	224	1,531	971	6,238

The proportion of out-of-pocket non-inpatient expenses showed minimal variation across different groups when compared to the total non-inpatient expense. A marginal decrease was observed in the high-expense group, while a slight increase was noted in the low-expense group. The proportion of outpatient expenses exhibits a more significant change after reimbursement when compared to the cost of medications at pharmacies. Compared to participants covered by URRMI, the expenditure of UEMIS demonstrates a more pronounced change after reimbursement. Given that UEMIS provides superior coverage for non-inpatient expenses than URRMI, the data suggests that participants covered by UEMIS demonstrate an enhanced capacity for payment, utilize more medical services, and incur higher costs. To sum up, the social health insurance schemes in China do not provide sufficient coverage for non-inpatient expenditures, particularly for URRMI (see Table 3).

The distribution of reimbursement rate for outpatient and pharmacies expenditure is skewed, thus quartiles were employed to describe it. According to the data presented in Table 4, a majority of enrollees did not receive reimbursement from the health insurance system. In comparison to URRMI, individuals enrolled in UEMIS obtained higher levels of reimbursement primarily through their individual accounts.

**Table 4:** The median, 25th percentile, and 75th percentile of the reimbursement rate for non-inpatient services among UEMIS and URRMI enrollee (%).

Cost categorization	Outpatient		Pharmacies	
	UEMIS	URRMI	UEMIS	URRMI
0–100	0 (0,0)*	0 (0,0)	0 (0,100)	0 (0,0)
100-	0 (0,33.33)	0 (0,27.27)	0 (0,70)	0 (0,0)
300-	0 (0,55)	0 (0,30)	0 (0,33.33)	0 (0,0)
500-	0 (0,80)	0 (0,9.49)	0 (0,62.5)	0 (0,0)
1,000-	22.22 (0,70)	0 (0,50)	0 (0,66.67)	0 (0,0)

^*^Median (p25, p75).

### Catastrophic health expenditure caused by non-inpatient cost

Before health insurance reimbursement, the prevalence of CHE among enrollee of UEMIS and URRMI was 20.52% and 36.24%, respectively. After reimbursement, these figures decreased to 14.26% and 25.06%, respectively, suggesting that UEMIS provides superior protection against non-hospitalization expenses compared to URRMI (Table 5).

**Table 5:** The incidence of CHE before and after reimbursement (n/%).

CHE	Before reimbursement		After reimbursement	
	UEMIS	URRMI	UEMIS	URRMI
Yes	374 (20.52)	4029 (36.24)	260 (14.26)	2786 (25.06)
No	1449 (79.48)	7090 (63.76)	1563 (85.74)	8333 (74.94)

The incidence of CHE was found to be significantly associated with various factors, including gender (male, female), age (under 60 years, 60–69, above 70 years), marital status (with and without spouse), education level (illiterate, primary school, middle school, college), place of residence (urban, rural), geographical area (eastern, central, western), per capita household expenditure [Quintile1 (lowest), Quintile2, Quintile3, Quintile4, Quintile5 (highest)], health insurance coverage(UEMIS, URRMI), self-reported health status (very good, good, fair, poor, very poor) and number of chronic conditions(none, one, two, three and more) (Table 6).

**Table 6:** The influential factors of CHE.

Characteristics	Without CHE (*n* (%))	With CHE (*n* (%))	χ^2^	*P*
Gender				
Male	4,357 (70.91)	1787 (29.09)	71.4707	<0.001
Female	4,346 (63.93)	2,452 (36.07)		
Age				
<60	4,677 (70.62)	1946 (29.38)	77.0574	<0.001
60–69	2,657 (64.87)	1,439 (35.13)		
> = 70	1,369 (61.58)	854 (38.42)		
Marriage				
With spouse	7,685 (67.68)	3,670 (32.32)	7.8919	0.005
Without spouse	1,018 (64.15)	569 (35.85)		
Education				
Illiterate	3,420 (63.7)	1949 (36.3)	125.0123	<0.001
Primary school	1869 (65.17)	999 (34.83)		
Middle school	3,179 (71.66)	1,257 (28.34)		
College	235 (87.36)	34 (12.64)		
Residency				
Urban	2,613 (72.54)	989 (27.46)	63.5777	<0.001
Rural	6,090 (65.2)	3,250 (34.8)		
Geographical area				
Eastern	3,146 (71.47)	1,256 (28.53)	92.9744	<0.001
Central	2,938 (68.21)	1,369 (31.79)		
Western	2,619 (61.87)	1,614 (38.13)		
Per capita household expenditure quintiles				
Quintile1 (lowest)	1,449 (55.97)	1,140 (44.03)	414.8943	<0.001
Quintile2	1,593 (61.55)	995 (38.45)		
Quintile3	1730 (66.85)	858 (33.15)		
Quintile4	1846 (71.33)	742 (28.67)		
Quintile5 (highest)	2085 (80.53)	504 (19.47)		
Health insurance				
UEMIS	1,425 (78.17)	398 (21.83)	114.9136	<0.001
URRMI	7,278 (65.46)	3,841 (34.54)		
Self-reported health status				
Very good	1,478 (87.4)	213 (12.6)	1,200	<0.001
Good	1,403 (82.38)	300 (17.62)		
Fair	4,139 (67.62)	1982 (32.38)		
Poor	908 (44.51)	1,132 (55.49)		
Very poor	203 (37.8)	334 (62.2)		
Numbers of chronic condition			486.7009	<0.001
None	5,698 (73.82)	2021 (26.18)		
One	2090 (62)	1,281 (38)		
Two	635 (53.54)	551 (46.46)		
Three and more	280 (42.04)	386 (57.96)		

The likelihood of experiencing CHE was significantly higher among participants with greater healthcare needs, such as those aged over 60 years and individuals with chronic diseases (*p* < 0.001). Furthermore, there was an inverse relationship between per capita household consumption expenditure and the probability of encountering CHE. Notably, the prevalence of CHE decreased from 44.03% among the most economically disadvantaged to 19.47% among the wealthiest participants.

### Key determinants of CHE (logistic model)

The logistic regression analysis results presented in Table 7 demonstrate the determinants of CHE. The model reveals a significant negative association between per capita household expenditure and CHE, indicating that as household expenditure increases, the likelihood of experiencing CHE decreases significantly (OR = 0.29–0.80, *p* < 0.001). Furthermore, individuals covered by URRMI exhibit a higher incidence of CHE compared to those covered by UEMIS (OR = 1.23, *p* = 0.02), emphasizing the crucial role of insurance coverage in mitigating financial burden. The incidence rate of CHE is 1.38 times higher in women compared to men, indicating a significantly elevated risk among the female population (OR = 1.34, *p* < 0.001). Moreover, individuals residing in the western region exhibit a 1.21-fold increased likelihood of experiencing CHE when compared to their counterparts living in the eastern area (OR = 1 0.31, *p* < 0 0.001).Higher health needs continue to be significant determinants of CHE. Older individuals with multiple chronic diseases and self-perceived poorer health are more likely to experience CHE. Specifically, individuals who rate their health as very poor have a 7.73-fold higher likelihood of experiencing CHE compared to those who rate their health as excellent (OR = 7.73, *p* < 0.001). Individuals with three or more chronic conditions have a 2.14-fold higher likelihood of experiencing CHE compared to those without any chronic illnesses (OR = 2.14, *p* < 0.001). The risk of CHE among individuals aged 70 and above is 1.24-fold higher compared to those under the age of 60 (OR = 1.24, *p* < 0.001).

**Table 7:** Determinants of catastrophic health expenditure: a logistic regression model.

Variables	Odds ratio	Std. err.	z	*P* > z	95% CI
Gender					
Male	Reference				
Female	1.341	0.065	6.020	<0.001	1.219,1.476
Marriage					
With spouse	Reference				
Without spouse	0.952	0.069	−0.680	0.498	0.825,1.098
Age					
<60	Reference				
60–69	1.104	0.059	1.860	0.063	0.994,1.227
> = 70	1.242	0.085	3.170	0.002	1.086,1.420
Education					
Illiterate	Reference				
Primary school	1.183	0.071	2.780	0.005	1.051,1.331
Middle school	1.108	0.068	1.650	0.098	0.981,1.250
College	0.952	0.229	−0.200	0.838	0.593,1.527
Geographical area					
Eastern	Reference				
Central	1.111	0.064	1.820	0.069	0.992,1.245
Western	1.314	0.075	4.780	<0.001	1.175,1.469
Residency					
Urban	Reference				
Rural	1.028	0.064	0.440	0.657	0.910,1.161
Health insurance					
UEMIS	Reference				
URRMI	1.233	0.111	2.320	0.020	1.033,1.471
Self-reported health status					
Very good	Reference				
Good	1.523	0.178	3.590	<0.001	1.211,1.915
Fair	2.751	0.264	10.560	<0.001	2.280,3.320
Poor	5.739	0.593	16.900	<0.001	4.686,7.028
Very poor	7.731	0.996	15.880	<0.001	6.007,9.951
Numbers of chronic condition					
None	Reference				
One	1.426	0.076	6.670	<0.001	1.284,1.582
Two	1.732	0.131	7.240	<0.001	1.493,2.010
Three and more	2.144	0.203	8.040	<0.001	1.780,2.582
Per capita household expenditure quintiles					
Quintile1 (lowest)	Reference				
Quintile2	0.803	0.053	−3.330	0.001	0.706,0.914
Quintile3	0.597	0.041	−7.450	<0.001	0.521,0.684
Quintile4	0.517	0.037	−9.170	<0.001	0.449,0.596
Quintile5(highest)	0.289	0.024	−14.880	<0.001	0.245,0.340
CON	0.074	0.011	−17.340	<0.001	0.055,0.100

### Limitation

The health financial risks induced by outpatient fees and pharmacy drug purchases were analyzed in this study based on social survey data. However, the fragmentation of these costs frequently impedes accurate recall by patients, and the survey only captured expenses incurred within the past month. The relevant calculations relied on monthly fees to estimate annual expenses, which may introduce certain biases. For countries lacking robust risk sharing mechanisms, particularly those with health insurance systems that prioritize hospitalization costs, it is imperative to pay attention to the health financial risk associated with non-inpatient expenses. The development of big data systems can facilitate obtaining comprehensive and accurate non-inpatient expenses data through various channels such as medical insurance platforms and hospital databases. This would significantly aid future analyses and comprehension of the health financial risk related to non-inpatient fees.

## Discussion

Since the establishment of the social health insurance scheme in China, coverage for non-inpatient fees has been limited. In the 1990s, outpatient fees were relatively low and health insurance funds primarily focused on mitigating risks associated with inpatient expenses. Nevertheless, with the advancements in clinical techniques along with changes in disease patterns, there has been a rapid increase in outpatient expenditures and a significant transformation health financial risks. For instance, a notable proportion of participants (10.44 and 7.93% enrolled in the UEMIS and URRMI schemes respectively) incurred monthly non-inpatient expenses exceeding 1,000RMB, while their annual costs for non-inpatient care surpassed the average hospitalization expenditure in 2021 (11002RMB) (28). This finding suggests that non-inpatient costs may be substantial.

Unfortunately, China’s social health insurance benefit package has not kept pace with these changes resulting in uncovered outpatient expense risks. Due to inadequate insurance coverage for outpatient expenses, many patients opt to purchase medications from pharmacies instead of seeking outpatient services from healthcare institutions as a cost-saving measure. In China healthcare institutions typically have their own pharmacies that offer medications alongside consultations, patients who are not eligible for reimbursement choose purchasing medications from pharmacies instead due to consultation fees charged by hospital. The data reveals a relatively low utilization rate of outpatient services compared to a relatively high utilization rate of pharmacy purchases.

The impact of non-inpatient medical expenses on CHE is substantial. Our analysis reveals that 14.26% of participants enrolled in the UEMIS and 25.06% of those covered by URRMI experienced CHE due to non-inpatient medical expenses. However, it is evident that social health insurance falls short in adequately protecting against non-inpatient cost related risks, as indicated by a reduction of only 6.25% and 11.18% in the proportion of participants experiencing CHE for UEMIS and URRMI beneficiaries, respectively.

The incidence of incurring CHE due to non-inpatient costs is positively associated with lower income level and poorer health status. Despite the partial coverage of outpatient expenses related to chronic diseases by China’s two basic health insurance systems, these policies offer limited benefits with stringent eligibility criteria. Consequently, a substantial number of patients with chronic diseases are unable to avail themselves of the advantages provided by health insurance policies, thereby facing significant financial health risk.

The present study revealed a statistically significant, albeit modest, elevated likelihood of CHE among individuals insured under the UEMIS compared to those insured under URRMS in China. There remains a disparity in the benefit package offered by China’s two fundamental health insurance schemes.

Despite the significant financial risk posed by non-inpatient costs, both governmental bodies and scholars have not given it adequate attention. While they continue to prioritize hospitalization expenses, they tend to overlook the issue of outpatient fees. The practices in certain provinces of China have demonstrated that reforming reimbursement policies pertaining to non-inpatient expenses will exert a substantial impact on the expenditure structure and efficacy of the health insurance fund. Therefore, it is imperative to underscore the necessity of devoting greater attention and conducting more comprehensive analysis regarding non-inpatient expenditures.

## Conclusion

Our study presents a novel body of evidence by demonstrating the financial risk associated with out-of-pocket expenses (OOPE) for non-inpatient care in China. The issue of financial risks linked to non-inpatient costs and their integration into health insurance benefit packages has reached a stage necessitating comprehensive analysis and inclusion on the government decision agenda.

Following the establishment of the National Health Security Administration in China in 2018, governmental efforts were made to incorporate specific non-inpatient fees, such as drug expenses for patients with hypertension and diabetes covered by URRMI, into the benefit package. However, these conservative reform measures fail to align adequately with the distribution of non-inpatient costs and associated risks, thereby impeding the achievement of policy objectives.

It is crucial to optimize the allocation of funds between inpatient and outpatient expenses by adjusting the benefit package, aiming to effectively manage health insurance fund expenditure. This paper seeks to garner governmental attention and prompt action on this pressing matter.

## Data Availability

Publicly available datasets were analyzed in this study. This data can be found at: https://charls.pku.edu.cn.
